# Polyoxometalates bind multiple targets involved in Alzheimer’s disease

**DOI:** 10.1007/s00775-025-02111-2

**Published:** 2025-03-22

**Authors:** Karin Ben Zaken, Rivka Bouhnik, Naama Omer, Naamah Bloch, Abraham O. Samson

**Affiliations:** Azrieli Faculty of Medicine, Bar Ilan University, Safed, Israel

**Keywords:** Polyoxometalate, Alzheimer’s disease, Molecular docking

## Abstract

Alzheimer's disease (AD) is a progressive neurodegenerative disorder characterized by brain aggregates of amyloid-β (Aβ) plaques and Tau tangles. Despite extensive research, effective therapy for AD remains elusive. Polyoxometalates (POMs), a class of inorganic compounds with diverse chemical structures and properties, are emerging as potential candidates for AD treatment due to their ability to target key molecular players implicated in disease pathogenesis, such as Aβ, acetylcholinesterase (AChE) and butyryl acetylcholinesterase (BChE). Here, we use molecular docking to predict the binding pose and affinities of POMs to 10 top targets associated with AD. First, we validate our method by replicating experimentally known binding of POMs to Aβ (Δ*G* = – 9.67 kcal/mol), AChE (Δ*G* = – 9.39 kcal/mol) and BChE (Δ*G* = – 10.86 kcal/mol). Then, using this method, we show that POM can also bind β-secretase 1 (BACE1, Δ*G* = – 10.14 kcal/mol), presenilin 1 (PSEN1, Δ*G* = – 10.65 kcal/mol), presenilin 2 (PSEN2, Δ*G* = – 7.94 kcal/mol), Amyloid Precursor Protein (APP, Δ*G* = – 7.26 kcal/mol), Apolipoprotein E (APOE4, Δ*G* = – 10.05 kcal/mol), Microtubule-Associated Protein Tau (MAPT, Δ*G* = – 5.28 kcal/mol) depending on phosphorylation, and α-synuclein (SNCA, Δ*G* = – 7.64 kcal/mol). Through such binding, POMs offer the potential to mitigate APP cleavage, Aβ oligomer neurotoxicity, Aβ aggregation, thereby attenuating disease progression. Overall, our molecular docking study represents a powerful tool in the discovery of POM-based therapeutics for AD, facilitating the development of novel treatments for AD.

**Graphical abstract:**

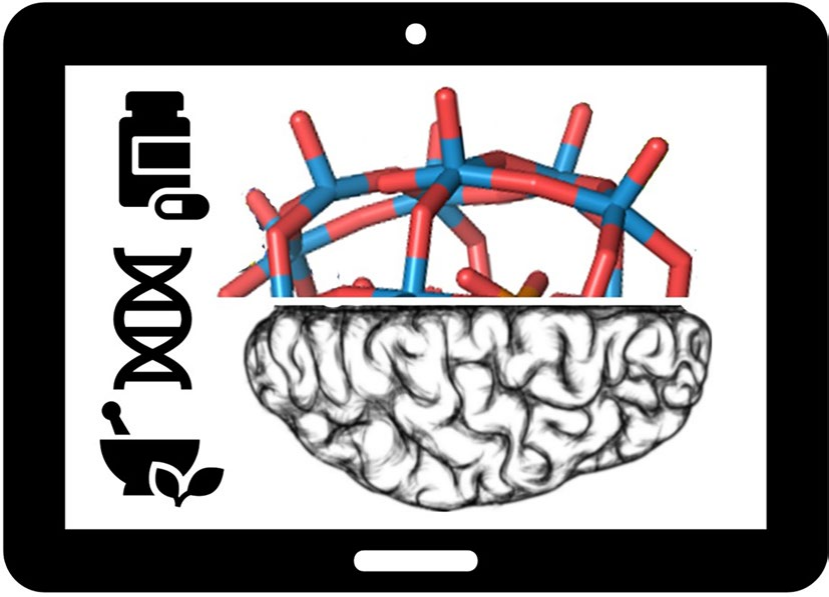

**Supplementary Information:**

The online version contains supplementary material available at 10.1007/s00775-025-02111-2.

## Introduction

Alzheimer’s disease (AD) represents a significant and growing global health concern, characterized by progressive cognitive decline and memory impairment. AD pathology is characterized by the accumulation of amyloid-β (Aβ) and Tau proteins in the brain, leading to the formation of insoluble plaques and tangles [1]. Aβ peptide is generated following the sequential cleavage of amyloid precursor protein (APP), by β-secretase (BACE1), and γ-secretase in the amyloidogenic pathway. Aβ genesis is precluded if APP is cleaved by α-secretase within the Aβ domain in the non-amyloidogenic pathway [2]. While Aβs aggregate into insoluble plaques, oligomeric species of amyloid remain bioactive and potentially drive neurodegeneration [3]. Tau is a microtubule-associated protein, that stabilizes microtubules for axonal transport. Tau phosphorylation by kinases, in particular at amino acids that interact with other sites that are phosphorylated in AD, reduces the affinity of tau for the microtubules [4]. Hyperphosphorylated tau aggregates into neurofibrillary tangles, while oligomeric species thereof could also remain bioactive.

Inhibition of the aforementioned enzymes leading to bioactive oligomeric states of Aβ and tau has emerged as a potential therapeutic strategy for AD [5]. Numerous small molecule inhibitors and other therapeutic agents have been developed and investigated for their ability to modulate AD proteopathies [6]. These inhibitors typically target the catalytic site, preventing enzymatic cleavage of APP by secretases, and subsequent generation of Aβ peptides; or tau phosphorylation by GSK-3β, p38 MAPK, CDK5, ERK1/2 and JNK3, and subsequent generation of hyperphosphorylated tau [7]. Over the past few decades, significant efforts have been made to identify and optimize such inhibitors with improved potency, selectivity, and pharmacokinetic properties. Current approved treatments of AD by the Food and Drug Administration (FDA) include acetylcholinesterase (AChE) inhibitors (i.e. donepezil, galantamine, rivastigmine), N-methyl-d-aspartate (NMDA) receptor inhibitors (i.e. memantine), and anti-amyloid monoclonal antibodies (i.e. lecanemab and aducanumab). However, treatment is mostly symptomatic and results in moderately less decline in cognition [8].

Polyoxometalates (POMs) are a large class of anionic metal–oxygen clusters that are built from early transition metals, bridged by oxygen atoms, and often including heteroatoms such as phosphorous or silicon [9]. POMs exhibit remarkable structural diversity, ranging from simple symmetric structures to highly complex architectures with diverse properties [10]. POMs are represented by the general formula [XxMmOo]^n−^, where X is a heteroatom, M is an early transition metal ion in the highest oxidation state, and are classified into different types, with the principal ones being Lindqvist [M_6_O_19_]^n−^, Keggin [XM_12_O_40_]^n−^, Wells–Dawson [XM_18_O_62_]^n−^, Silverton [XM_12_O_42_]^n−^, Preyssler [X^n+^P_5_W_30_O_110_]^(15−n)−^ and Anderson-Evans [XM_6_O_24_]^n−^ types [11]. Lacunary POMs are derived from the removal of one or more metal centers from a parent POM structure, creating holes or vacancies. These vacancies make lacunary POMs highly reactive and useful as building blocks for larger, more complex structures or for coordination with other metal ions or organic molecules.

In recent years, POMs have garnered attention for their potential biomedical applications [12–14]. Research suggests that certain POMs exhibit antibacterial [15], antiviral [16], and anticancer [17] properties. POMs form interactions with multiple proteins, mainly through salt-bridges and hydrogen bonds with positively-charged, and polar residues [18]. For example, POMs bind to DNA polymerases, and putatively mimic DNA interactions [19]. Likewise, POMs bind to Sox2, and competitively inhibit DNA binding [20]. In addition, POMs inhibit protein-kinase CK2 [21], and Actomyosin ATPase [22]. Indeed, POMs are superchaotropes, that form high affinity interactions with multiple proteins in aqueous solutions [23]. As such, POM could resolve protein aggregation making them promising candidates for therapeutic interventions also in the treatment of AD [24].

In a seminal study, Geng et al. have shown that POMs bind to Aβ peptides with micromolar affinity [25]. The main force driving the interaction is an electrostatic attraction between the negatively charged POMs and the positively charged H13–K16 cluster region of Aβ [25]. Most recently, Ma et al. reviewed the potential of POM in combating Aβ aggregation, as well as the latest progress using POMs to mitigate AD [26]. In another important study, Bondžić et al. have found that POMs bind to AChE with nanomolar affinity [27]. Molecular docking experiments have revealed that the negatively charged POM binds to an allosteric site located at the entrance of the positively charged gorge of AChE, thereby occluding substrate entry into the catalytic site. As such, POM is also an inhibitor of the enzymatic activity of AChE. Lastly, Iqbal et al. have also found that POMs bind to AChE, and butyrylcholinesterase (BChE) with nanomolar affinity [28]. Notably, POMs traverse the blood–brain barrier (BBB) [29, 30].

Here, we use molecular docking and predict POM binding to protein targets associated with AD. First, we validate our docking method, and replicate the experimental POM binding to Aβ, to AChE. Then, we perform a set of molecular dockings and predict POM binding to 10 top protein targets associated with AD.

## Methods

### Top protein targets associated with AD

To identify the top protein targets associated with AD, we used a list of 19,834 protein-coding human genes obtained from NCBI Gene. For each gene name, we counted the number of citations returned in a PubMed search for the query: (“gene name”) AND (“Alzheimer’s disease”) and divided it by the number of citations returned in a PubMed search for the query: (“gene name”) to normalize association. This process was automated using an in-house Python script. The Python script retrieved the number of citations from PubMed using the command ‘wget’, and helped rank protein targets according to the normalized association with AD.

### PDB structure selection

To select a representative PDB structure of the top protein targets associated with AD, the PDB was queried using the protein name, a human sequence, a complete chain, and the best X-ray resolution. For each PDB structure, ligands and water molecules, but not metal ions were edited out before further analysis**.**

### Molecular docking

To perform molecular docking, and predict POM binding to protein targets associated with AD, we used AutoDock Vina [31]. For each protein structure, a diverse set of POM structures was selected. POM and protein structures were converted to the PDBQT format using AutoDock Tools, ensuring appropriate atom labeling and types. Grid parameter files (.gpf) were prepared to define the search space (grid box) around the active site for molecular docking simulations. The coordinates and dimensions of the grid box were determined based on the location and size of the active site residues, as identified from available structural data. If no active site data were available, then the coordinates and dimensions of the grid box were set to encompass the whole protein with a 2 Å margin in x, y, and z, directions. Table 1 lists the grid box parameters of the catalytic site used for each target.

**Table 1:** Lists of AutoDock grid parameters used for each protein

		Grid center (Å)			Grid size (Å)		
Protein	PDB	x	y	z	x	y	z
APP	2fjz	13.2	15.0	5.4	80	104	96
APOE4	6nco	– 6.5	– 11.3	21.2	96	69	122
BACE	2g94	28.7	41.0	39.0	120	126	112
PSEN1	7y5t	168.8	173.9	158.6	126	126	126
PSEN2	7y5x	110.4	109.3	93.1	92	126	118
ACHE	1c2b	23.6	83.2	22.8	126	126	126
BCHE	4tpk	– 4.1	8.8	16.8	104	118	106
MAPT	5e2w	32.2	– 1.6	– 6.3	64	46	40
BDNF	1bnd	13.7	– 11.6	45.9	126	106	126
SNCA	1xq8	233.8	60.6	– 15.1	74	126	90
Aβ	2lfm	3.1	– 7.5	– 17.7	84	108	84

In addition, parameter files (.dpf) were prepared for AutoDock simulations, defining the search space around the active site and specifying docking parameters. Molecular docking simulations were conducted employing the prepared PDBQT files of POM ligands, and parameter files. Docking calculations were performed to predict binding modes and affinities of POMs in the active site. The binding constant, K_d_, was calculated using the formula K_d_ = ${e}^{-\frac{\Delta G}{RT}}$, in which $\Delta G$ is Gibbs free binding energy (kcal/mol), R is the gas constant 0.00198 kcal/mol, and T at room temperature is 298°K. Interactions and binding energies of docked POMs were visually inspected using PyMol (Schrödinger).

## Results

### Top protein targets associated with Alzheimer’s disease (AD)

The top 10 associations of AD with 19,834 genes are listed in Table 2. The top 50 protein targets associated with AD are listed in Supplementary Table 1. The top proteins are well-known to be linked with AD, and have served as targets in computer-aided drug design.

**Table 2:** Top associated genes with AD

Rank	Gene	Name	Normalized association
1	APP	Amyloid Precursor Protein	89%
2	APOE	Apolipoprotein E	83%
3	PSEN1	Presenilin 1	80%
4	MAPT	Microtubule Associated Protein Tau	79%
5	BACE1	β-secretase 1	78%
6	SNCA	α-synuclein	73%
7	PSEN2	Presenilin 2	72%
8	BDNF	Brain-derived neurotrophic factor	71%
9	ACHE	Acetylcholinesterase	71%
10	BCHE	Butyrylcholinesterase	70%

### Validation

AutoDock Vina is a widely used molecular docking software tool for predicting the binding modes and affinities of small molecules to target proteins [31]. It employs an efficient search algorithm and scoring function to explore the conformational space of ligands and predict their optimal binding poses within the binding site of a protein.

To validate the use of AutoDock Vina molecular docking with the inorganic ligand, polyoxometalate (POM), we first attempted to replicate the experimental dissociation constant of POM [P_2_NiW_17_O_61_]^−8^ with Aβ, K_d_ = 0.56 μM (Δ*G* = – 7.7 kcal/mol) measured by Gao et al. [29](Supplementary Table 2). Remarkably, our predicted average affinity of POM [P_2_NiW_17_O_61_]^−8^ (modified PDB 5fhw) to Aβ (PDB 2lfm) is Δ*G* = – 9.67 kcal/mol. Moreover, our prediction is in agreement with the reported electrostatic attraction between the negatively charged POMs and the positively charged H13–K16 cluster region of Aβ [25]. Also, the experimental dissociation constant of another POM [β-SiW_11_O_39_]^8−^ with Aβ, K_d_ = 2.1 μM (Δ*G* = – 8.45 kcal/mol) measured by Geng et al. [25], is close to the predicted mean using the POM [ZrPW_11_O_39_]^3−^ (PDB 4xyy) bound to Aβ (PDB 2lfm) Δ*G* = – 8.79 kcal/mol.

Then, we attempt to replicate the experimental binding affinity of POM [SiW_11_O_39_]^7−^ to AChE, IC_50_ = 72.3 nM (Δ*G* ≈ – 9.7 kcal/mol) measured by Bondžić et al. [27](Supplementary Table 2). The predicted mean affinity of POM [SiW_11_O_39_]^7−^ (modified PDB 6y7o) to AChE (PDB ID 1c2b) is K_d_ = 159.4 nM (Δ*G* = – 9.39 kcal/mol). Assuming competitive inhibition, then IC_50_ = K_d_(1 +  [S]/K_m_). If [S] is ~ 10 μM and K_M_ ~ 10 μM, then IC_50_ = K_i_ *2 is expected, and IC_50_ = 72.3 nM and K_d_ = 159.4 nM are in agreement with the assumption. In any case, the IC_50_ and K_d_ are similar enough in magnitude (nM) for the purpose of validation.

Finally, we attempt to replicate the experimental binding affinity of POM [TeW_6_O_24_]^−6^ to BChE, IC_50_ = 1560 nM (Δ*G* ≈ – 7.88 kcal/mol) determined by Iqbal et al. [28] (Supplementary Table 2). The predicted mean affinity of POM [TeW_6_O_24_]^−6^ (PDB 7ovs) to BChE (PDB 4tpk) is K_d_ = 0.73 nM (Δ*G* = – 10.86 kcal/mol). Assuming competitive inhibition, the values diverge by orders of magnitude.

**Fig. 1:**
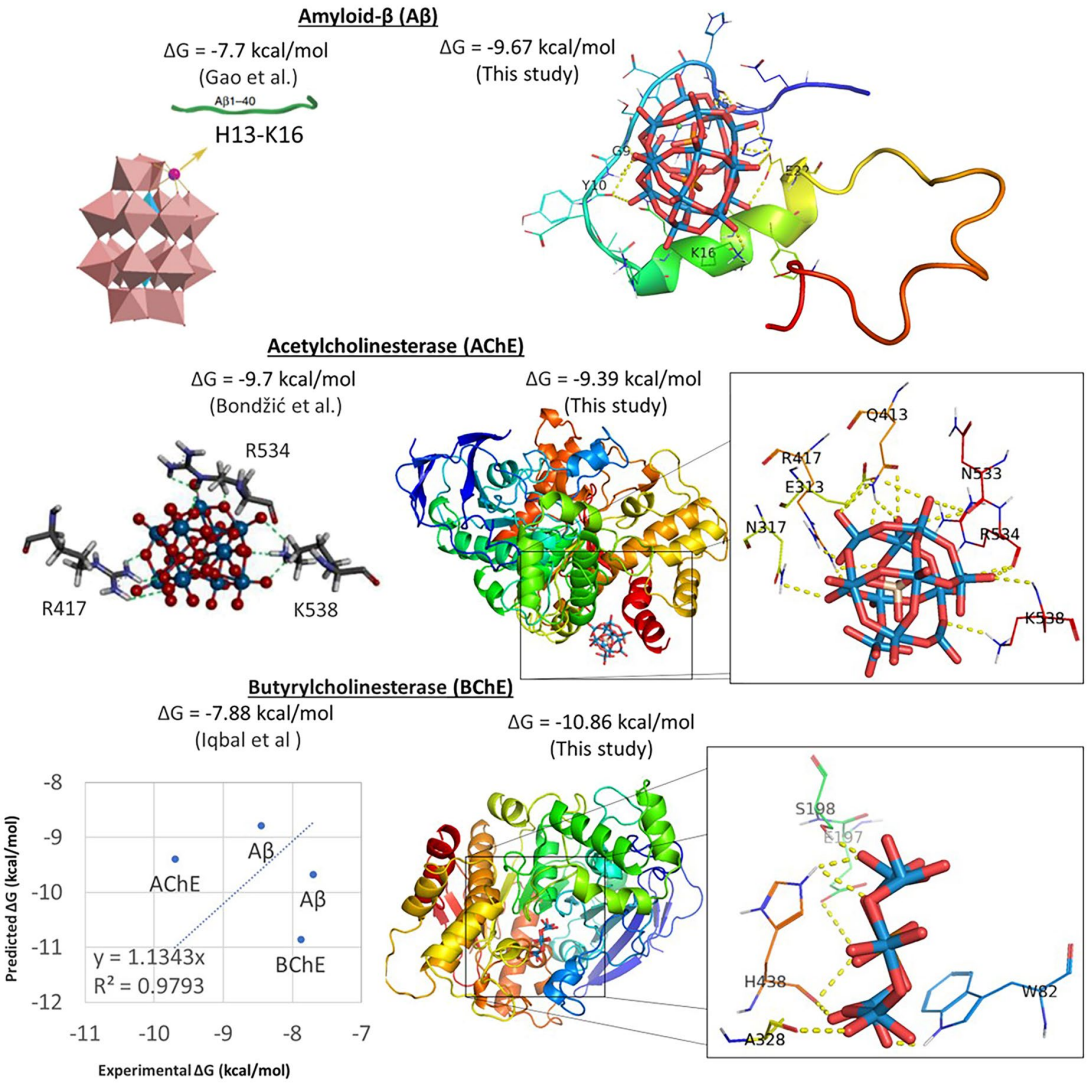
Molecular docking validation. On top left is the experimental binding affinity of POM [P_2_NiW_17_O_61_]^−8^ to Aβ [29]. On top right is our predicted pose of POM ( [P_2_NiW_17_O_61_]^−8^, modified PDB 5fhw) interacting with Aβ (PDB 2lfm). On the middle left is the predicted pose of POM SiW_11_O_39_]^7−^ in the allosteric binding site of AChE [27]. On the middle right is our predicted pose of POM ( [SiW_11_O_39_]^7−^, modified PDB 6y7o) in the allosteric binding site of AChE (PDB 1c2b). Note that the presumed interactions are similar. On the bottom right is our predicted pose of POM ( [TeW_6_O_24_].^−6^, PDB 7ovs) in the binding site of BChE (PDB 4tpk), next to the experimental binding affinity [28]. On the bottom left is a plot of the predicted and experimental binding energies (Δ*G*), showcasing a correlation of 0.98

However, taken together, all the predicted binding values are close enough to the experimental one, and validate our molecular docking technique with POMs. The correlation coefficient between the experimental predicted binding energies is R^2^ = 0.979 (Fig. 1, bottom left panel). Moreover, Fig. 1 shows that the predicted binding pose of POM with Aβ agrees with the interaction of POM with the H13 to K16 (HHQK) cationic cluster of Aβ, as proposed by Geng et al. [25], and that POM interacts with residues located at the entrance of the AChE gorge, as suggested by Bondjic et al. [27]. Figure 1 also shows that POM interacts with residues in the catalytic site of BCHE, as proposed by Iqbal et al. [28]. As such, our predicted binding affinities and binding interactions of POM with Aβ, AChE, and BChE validate of molecular docking approach.

### Predictions

**Fig. 2:**
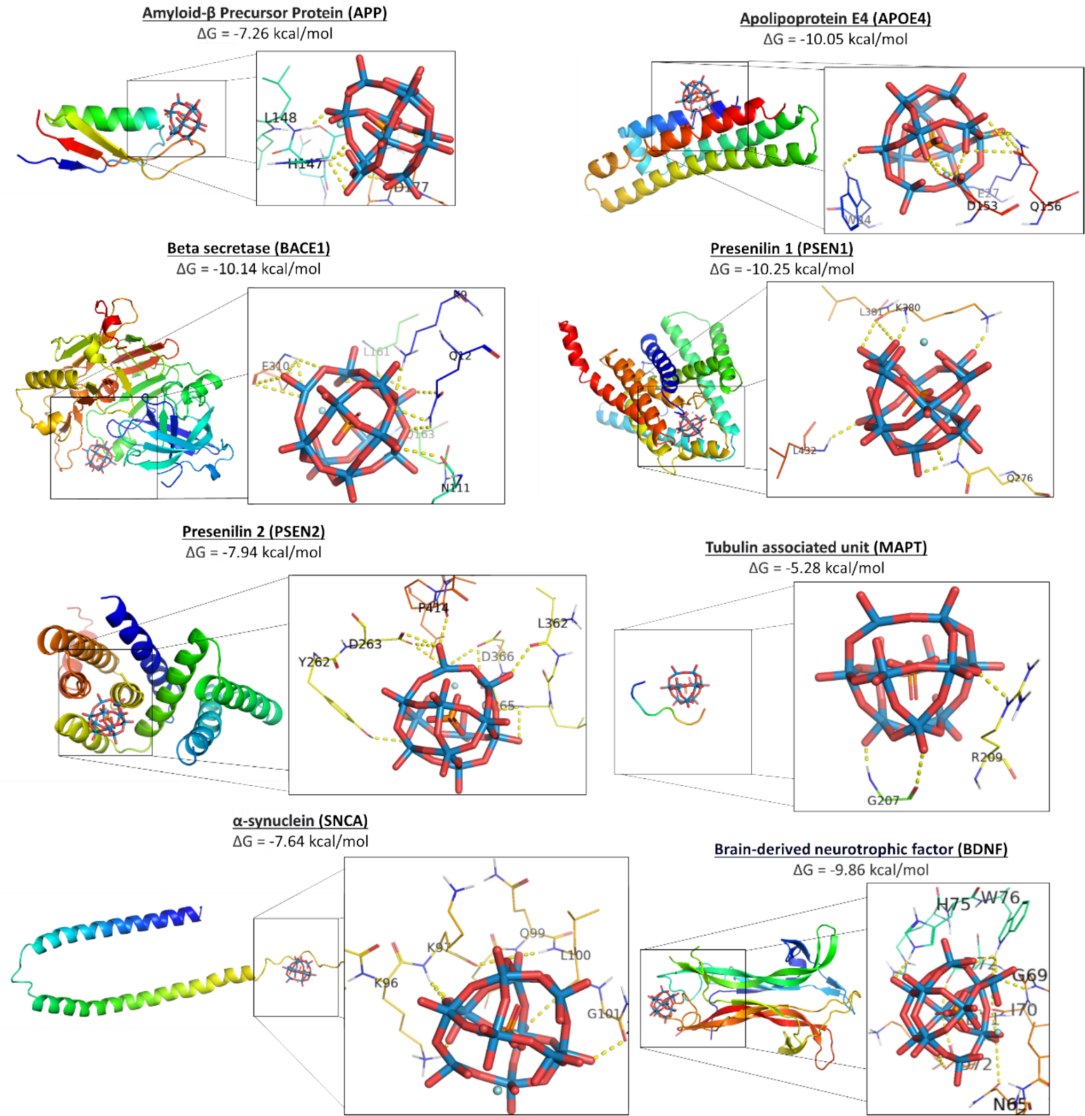
Molecular docking of POM [ZrPW_11_O_39_].^−3^ to top 8 protein targets of AD. Shown are the predicted pose of POM in the target binding site (left), and the interaction formed with individual residues (right). Key residues within the binding site are annotated, illustrating the specific interactions between POM (PDB 4xyy) and the protein targets APP (PDB 2fjz), APOE4 (PDB 6nco), BACE1(PDB 2g94), PSEN1 (PDB 7y5t), PSEN2 (PDB 7y5x), MAPT (PDB 5e2w), SNCA (PDB 1xq8), and BDNF (PDB 1bnd)

Having validated our molecular docking setup, we assessed the binding affinity of POMs to the protein linked with AD. Figure 2 depicts the three-dimensional complex formed between POM ( [ZrPW_11_O_39_]^−3^, PDB 4xyy) and protein targets associated with AD, highlighting the binding interactions. Figure 2 also shows the predicted binding affinity.

POM binds to the copper-binding domain of APP (mean Δ*G* = – 7.26 kcal/mol), and forms interactions with residues H147, H151, and Y168, similar to those formed by the copper ion [32]. The copper binding domain consists of residues 133–189 (PDB ID 2fjz), and POM mimics the metal interactions. As such, POM can potentially replace the copper ion, and provide conformational stability to the copper-binding domain of APP.

POM strongly binds to ApoE4 (mean Δ*G* = – 10.05 kcal/mol), and forms interactions with hydrophilic residues R35, and Q156. POM does not bind to C112 and C158, which are different in APOE variants. ApoE3 contains a mutation of C112R, while ApoE2 contains both mutations C112R and C158R. Of the three variants, ApoE4 has the most damaging effects on neurons, ApoE3 has intermediate effects, and ApoE2 is the most neuroprotective variant associated with AD [33]. ApoE4 is more potent and ApoE2 less potent than ApoE3 in stimulating Aβ production. POM does not bind adjacently to the free cysteines, and therefore should not affect APOE binding to the APOE receptor [34]. Potentially, POM could help solubilize low-density lipoproteins (LDL).

POM binds with BACE1 (mean Δ*G* = – 10.14 kcal/mol), and forms electrostatic interactions and hydrogen bonds with residues K21, T232, N233, and Q73 at the entrance of the active site domain, and prevents access to the active site domain. POM occludes the active site residues D32 and D228 of BACE1, and potentially prevents APP cleavage by β-secretase [35].

POM strongly binds to PSEN1 (mean Δ*G* = – 10.65 kcal/mol) and forms interactions with residues L49 D257, and D385 of the active site. These residues are part of the active site of PSEN1, and mutations thereof prevent activation of the intramembrane γ-secretase complex and APP cleavage [36]. As such, POM occludes the catalytic site of PSEN1, and potentially prevents APP cleavage by γ-secretase [37].

POM binds with PSEN2 (mean Δ*G* = – 7.94 kcal/mol), and forms in interactions with residues of the active site residues, D263 and D366 [38]. As such, POM occludes the catalytic site, and potentially prevents APP cleavage by γ-secretase.

POM weakly binds to MAPT (mean Δ*G* = – 5.28 kcal/mol) and forms interactions with R209. It does not form interactions with the phosphorylated residues of MAPT. Moreover, POM binds differently to each phosphorylation state of MAPT, and in some cases POM does not bind at all. As such, POM differential binding to MAPT variants is difficult to interpret in the context of AD.

POM binds to SNCA (mean Δ*G* = – 7.64 kcal/mol), and forms electrostatic interactions with K65, K97, and Q99 of α-synuclein. While α-synuclein is more associated to Parkinson’s disease, than to AD, it also co-aggregates with Aβ [39]. POM binds to α-synuclein, (in various positions) and potentially solubilizes it. As such, POM has a benign effect on SNCA in the context of AD.

POM strongly binds to BDNF dimers (mean Δ*G* = – 9.86 kCal/mol), and stabilizes this construct which binds to TrkB tyrosine kinase receptor [40]. As such, POM potentially improves neuronal function, and prevents cell death.

POM strongly binds to AChE and BCHE. POM binds at an allosteric site, at the opening of the gorge leading into the catalytic site. POM occludes the active site, and prevents catalytic breakdown of acetylcholine. As such, POM potentially improves cholinergic dysregulation associated with AD.

The POM of focus in this study is a Zr^IV^‐substituted Keggin polyoxometalate with the chemical formula [ZrPW_11_O_39_]^−3^ (PDB ID 4xyy) [41]. It was chosen because of its well-defined 3D structure, and the availability of its molecular coordinates in the PDB. In addition, this Keggin POM has shown promising potential in earlier studies associated with AD, such as that of Geng et al. [25] and Bondžić et al. [27].

### POM variants

In all our predictions, we utilized the representative POMs previously described [25]. To expand the POM repertoire, we explored additional candidates of this inorganic compound in pursuit of even more negative values. Various POM structures were docked onto BACE1 (Table 3). This enzyme was chosen because it catalyzes the first step in the cascade leading to Aβ aggregation. Table 3 presents the results of molecular docking between POM and BACE1. Only negative values of free binding energy were obtained in molecular docking for POM and BACE1, indicating favorable interactions. Table 3 reveals a range of minimal binding affinities from – 9.72 to – 10.60 kcal/mol. Notably, POMs that contain transition metals, in particular nickel and cobalt, are quite toxic [42], and were not screened against other proteins associated with AD. Finally, the unsubstituted Keggin POMs most negatively charge exhibits the highest affinity towards BACE1.

**Table 3:** Predicted affinity of POM variants to BACE1

POM formula	PDB source	BACE1 affinity Δ*G*)kcal/mol(
[ZrPW_11_O_39_]^3−^	4xyy [41]	– 10.14
[ZnPW_11_O_39_]^5−^	6ruw [43]	– 10.48
[NiPW_11_O_39_]^5−^	6ruh [43]	– 10.14
[NiSiW_11_O_39_]^6−^	7a9m [44]	– 9.91
[CoSiW_11_O_39_]^6−^	7a9k [44]	– 9.72
[SiW_11_O_39_]^7−^	6y7o [45]	– 10.60

## Discussion

Our molecular docking simulations indicate that POM binding to Aβ and AChE is consistent with experimental findings. Moreover, the predicted POM-binding affinities and poses are similar to those reported by Geng et al. [25], Bondzic et al. [27], Iqbal et al. [28], and Gao et al. [29]. As such, these findings attest to the validity of our molecular docking simulations, and endorse further investigation using other protein targets linked with AD, and various POM compounds.

Remarkably, our molecular docking simulations suggest that POM binds to all 10 protein targets of AD with various degrees of affinity. In order of potency, POM strongly binds to AChE (mean Δ*G* = – 9.39 kcal/mol), and to BChE (mean Δ*G* = – 10.86 kcal/mol), thus potentially improving cholinergic dysregulation associated with AD. POM strongly binds with BACE1 (mean Δ*G* = – 10.14 kcal/mol), and potentially prevents APP cleavage by β-secretase. POM strongly binds to ApoE4 (mean Δ*G* = – 10.05 kcal/mol), and potentially solubilizes low-density lipoproteins (LDL). POM strongly binds to PSEN1 (mean Δ*G* = – 10.65 kcal/mol) and PSEN2 (mean Δ*G* = – 7.94 kcal/mol), thus potentially preventing APP cleavage by γ-secretase. POM strongly binds to the bioactive BDNF dimers (mean Δ*G* = – 9.86 kcal/mol), and potentially improves neuronal function, and prevents cell death. POM binds to SNCA (mean Δ*G* = – 7.64 kcal/mol), and potentially solubilizes it. POM binds to the copper-binding domain of APP (mean Δ*G* = – 7.26 kcal/mol), and potentially stabilizes its native conformation. Finally, POM weakly binds to MAPT (mean Δ*G* = – 5.28 kcal/mol), however, little to no effect is expected. Another goal of this study has been to use molecular docking and evaluate the potential of diverse POMs. For this purpose, we docked an array of POM structures to BACE1. Through computational simulations, we have shown that most POM candidates exhibit similar affinities toward BACE1, thus offering alternative insights into potential avenues for AD therapy.

**Fig. 3:**
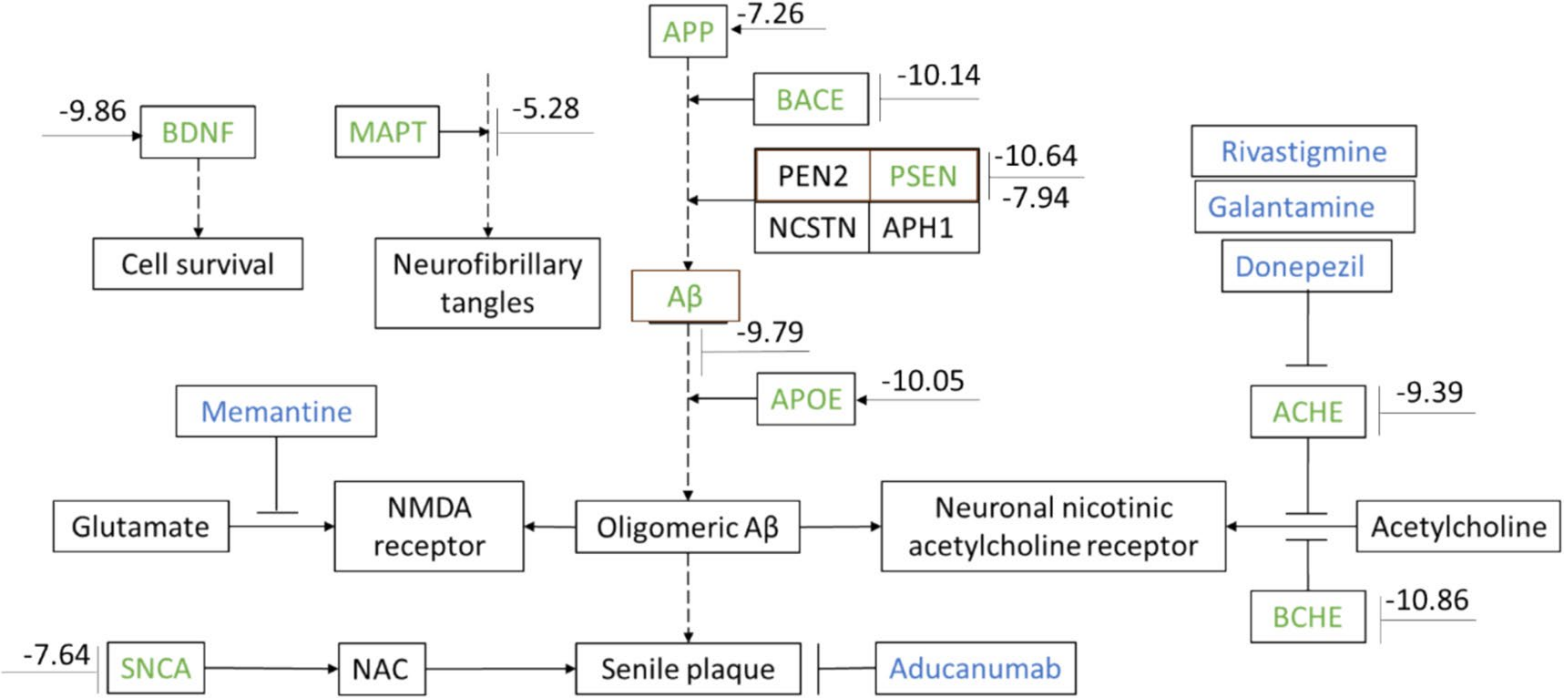
Pathway of top 10 genes involved in Alzheimer’s disease (AD). Shown in green are the top 10 genes associated with AD. Most are involved in amyloid β (Aβ) formation, like APP (amyloid precursor protein), BACE1 (Beta-Secretase 1), PSEN (Presenilin) 1 and 2, APOE (Apolipoprotein E), AChE (acetylcholinesterase), BChE (butyrylcholinesterase), and SNCA (α-synuclein). MAPT (Microtubule Associated Protein Tau) leads to the formation of neurofibrillary tangles. BDNF (Brain-derived neurotrophic factor) is involved in cell survival. FDA drugs approved for the treatment of AD are colored blue. Stippled and full lines show binding and product respectively. Numbers indicate predicted POM affinity; less than – 8 kcal/mol is considered strong binding, between – 8 and – 6 kcal/mol is deemed medium binding, and above – 6 kcal/mol is considered weak binding

To better visualize the POM effect, Fig. 3 shows the top protein targets associated with (AD) classified into pathways. Most are involved in the formation of Aβ peptides. First, APP is digested by BACE1, PSEN1, and PSEN2 to form Aβ. Then, Aβ binds to APOE and SNCA to form Aβ oligomers, aggregates, and senile plagues, that is partially alleviated by the FDA-approved drug aducanumab. Aβ also binds to nicotinic acetylcholine receptors [3], and depletes cholinergic neurotransmission by acetylcholine. AChE and BChE cleave acetylcholine, and deplete cholinergic transmission, which is partially restored by FDA-approved drugs donepezil, rivastigmine, and galantamine. Notably, Aβ also binds to NMDA receptors, and depletes excitatory neurotransmission by glutamate, which is partially restored by the FDA-approved drugs memantine. MAPT leads to formation of neurofibrillary tangles. BDNF is involved in cell survival. These highly associated pathway genes are related to AD, and expose the most prominent genes currently associated with this nefarious neurodegenerative disease.

The multifunctional nature and tunable properties of POMs, make them promising candidates in the treatment of AD. Notably, multiple studies have shown that POM are inhibitors of Aβ aggregation [25, 46–49]. Our molecular docking shows that POMs bind to cationic residues of Aβ, and potentially inhibit Aβ aggregation, thus reducing the cytotoxicity induced by Aβ. Also, POMs modified with peptides can suppress Aβ aggregation [50]. Notably, POMs consist of complex metal ions cages, can quench radicals, and serve as reducing agents to clean reactive oxygen species (ROS) common in AD [51]. The intrinsic charge carrier conduct also contributes to other applications of POM-based materials [52]. In addition, POM are potent inhibitors of AChE and BChE and have been proposed as potential drugs to treat AD [28]. Toxicity studies have shown that Keggin-type POMs are usually well tolerated with little to no toxicity [28], and show minimal hepatoxicity that is reversible [53]. Importantly, some of the POM used in this study, pass through the BBB, and enter the CNS [29, 30]. Moreover, BBB integrity is often compromised in AD patients [54]. Interestingly, the negatively charged unsubstituted POM, [SiW_11_O_39_]^7−^, displays the strongest affinity towards BACE1 (see Table 3). If the negative charge is neutralized by positively charged metal-ion substitutions, then the affinity of POM to positively charged binding surfaces decreases. Moreover, metal ions are sometimes toxic, and Ni and Co substituted POMs display better affinities to BACE1 (Table 3). Altogether, these properties make POMs attractive candidates for modulating AD and disrupting Aβ peptide generation.

### Limitations

As a potential limitation of this study, molecular docking is not an experimental assay. Molecular docking is a computational tool, that has gained prominence in drug discovery and design for its ability to predict the binding modes and affinities of small molecules to target proteins [31]. By simulating the interactions between ligands and receptors at the atomic level, molecular docking enables the identification of potential drug candidates and elucidation of their mechanisms of action. Importantly, our past predicted data [55], has been corroborated on several occasions [56]. Nevertheless, our theoretical data must be corroborated with experimental data.

As a potential limitation of this study, POM could interact through electrostatic interactions, and bind non-selectively to all proteins. To test this potential fallacy, we tested POM binding to random proteins. POM was docked to arginase (PDB ID 1t4s, – 4.03 kcal/mol), superoxide dismutase (PDB ID 1ba9, – 8.97 kcal/mol), and glutathione reductase (PDB ID 1dnc, – 11.92 kcal/mol), and found to vary greatly, ranging from binding to non-binding binding affinities. In fact, POMs are promiscuous protein binders, and their lack of selectivity has been associated with potential toxicity [57]. To reduce toxicity, POM selectivity has been harnessed, for example through hybridization with organic ligands [58]. Such hybrid POMs functionalized with organic moieties have shown promising results in finetuning POM selectivity. This study did not use functionalized POMs, and did not explore their infinite structural diversity.

## Conclusion

Our findings support the notion that POMs hold promise as potential inhibitors of 10 protein targets associated with Alzheimer’s disease. The similarity in binding affinities across different inhibitors suggests a common mechanism of action and highlights the potential for structural optimization to enhance inhibitory potency. Further investigation into the design and synthesis of POM-based inhibitors could lead to the development of novel therapeutic agents for AD.

## Electronic Supplementary Material

Below is the link to the electronic supplementary material.

Supplementary Material 1

## Data Availability

No datasets were generated or analysed during the current study.

## Abbreviations

AChE: Acetylcholinesterase
AD: Alzheimer’s disease
Aβ: Amyloid β
APOE: Apolipoprotein E
APP: Amyloid Precursor Protein
BBB: Blood–brain barrier
BACE1: β-Secretase 1
BChE: Butyrylcholinesterase
BDNF: Brain-derived neurotrophic factor
FDA: Food and Drug Administration
MAPT: Microtubule Associated Protein Tau
NMDA: N-methyl-d-aspartate
POM: Polyoxometalates
PSEN: Presenilin
ROS: Reactive oxygen species
SNCA: α-Synuclein
