# Supplementary material for: Polyoxometalates bind multiple targets involved in Alzheimer’s disease
Source: J Biol Inorg Chem. 2025 Mar 22;30(3):299–309. doi: 10.1007/s00775-025-02111-2 (PMC11965166; doi:10.1007/s00775-025-02111-2)
Supplement: Supplementary file 1 — Supplementary Material 1 [file 775_2025_2111_MOESM1_ESM.docx]

**Supplementary table 1.** List of top 50 genes associated with Alzheimer’s disease.

| Rank | Gene | Normalized Association (%) |
| --- | --- | --- |
| 1 | APP | 89 |
| 2 | APOE | 83 |
| 3 | PSEN1 | 80 |
| 4 | MAPT | 79 |
| 5 | BACE1 | 78 |
| 6 | SNCA | 73 |
| 7 | PSEN2 | 72 |
| 8 | BDNF | 71 |
| 9 | ACHE | 71 |
| 10 | BCHE | 70 |
| 11 | TREM2 | 66 |
| 12 | IL1B | 66 |
| 13 | NGF | 65 |
| 14 | CDK5 | 65 |
| 15 | YWHAQ | 64 |
| 16 | IDE | 64 |
| 17 | DLG4 | 64 |
| 18 | CHAT | 64 |
| 19 | AIF1 | 63 |
| 20 | IL6 | 63 |
| 21 | HTT | 63 |
| 22 | CLU | 62 |
| 23 | GFAP | 62 |
| 24 | SORL1 | 62 |
| 25 | NCSTN | 62 |
| 26 | TNF | 62 |
| 27 | CASP3 | 62 |
| 28 | PICALM | 61 |
| 29 | CREB1 | 61 |
| 30 | AKT1 | 61 |
| 31 | INS | 61 |
| 32 | ADAM10 | 60 |
| 33 | RBFOX3 | 60 |
| 34 | SYP | 60 |
| 35 | GRIN2B | 60 |
| 36 | APH1A | 60 |
| 37 | ACTB | 60 |
| 38 | ABCA7 | 59 |
| 39 | PSENEN | 59 |
| 40 | SOD1 | 59 |
| 41 | PRNP | 59 |
| 42 | NEFL | 59 |
| 43 | ALB | 59 |
| 44 | TARDBP | 58 |
| 45 | MAOB | 58 |
| 46 | BIN1 | 58 |
| 47 | GAPDH | 58 |
| 48 | IAPP | 58 |
| 49 | SIRT1 | 58 |
| 50 | APBB1 | 58 |

**Supplementary table 2.** Experimental binding affinities of polyoxometalates to AChE, BChE, and Aβ.

| Target | POM | IC50 | Kd ($\frac{1}{K_{a}}$) | Reference |
| --- | --- | --- | --- | --- |
| Aβ | [PTi_2_W_10_O_40_]7- | 39.04 μM | 2.15 μM | [25] |
| Aβ | [SiW_9_O_34_]^9-^ | 19.85 μM | 220 nM | [25] |
| Aβ | [β-SiW_11_O_39_]^8-^ | 39.02 μM | 2.10 μM | [25] |
| Aβ | [P_2_CoW_17_O_61_]^8-^ | 16.68 μM | 480 nM | [25] |
| AChE | [SiW_11_O_39_]^7−^ | 72.3 ± 0.2 nM | - | [27] |
| AChE | [PW_9_O_34_]^11−^ | 1230 ± 10 nM | - | [27] |
| AChE | [H_2_W_12_O_42_]^10−^ | 0.29 ± 0.01 μM | - | [28] |
| AChE | [TeW_6_O_24_]^6−^ | 0.31 ± 0.01 μM | - | [28] |
| BChE | [(O_3_PCH_2_PO_3_)_4_W_12_O_36_]^16−^ | 0.18 ± 0.05 μM | - | [28] |
| AChE | [H_2_W_12_O_40_]^6−^ | 2.30 ± 0.44 μM | - | [28] |
| BChE | [H_2_W_12_O_40_]^6−^ | 1.56 ± 0.46 μM | - | [28] |
| AChE | [P_6_W_18_O_79_]^20−^ | 8.71 ± 0.6 μM | - | [28] |
| BChE | [P_6_W_18_O_79_]^20−^ | 1.71 ± 0.82 μM | - | [28] |
| AChE | [P_8_W_48_O_184_]^40−^ | 4.47 ± 0.2 μM | - | [28] |
| BChE | [P_8_W_48_O_184_]^40−^ | 0.52 ± 0.04 μM | - | [28] |
| AChE | [(O_3_POPO_3_)_4_W_12_O_36_]^16−^ | 3.51 ± 1.84 μM | - | [28] |
| BChE | [(O_3_POPO_3_)_4_W_12_O_36_]^16−^ | 0.18 ± 0.05 μM | - | [28] |
| AChE | [(O_3_PCH_2_PO_3_)_4_W_12_O_36_]^16−^ | 5.04 ± 1.06 μM | - | [28] |
| BChE | [(O_3_PCH_2_PO_3_)_4_W_12_O_36_]^16−^ | 0.18 ± 0.05 μM | - | [28] |
| AChE | [H_2_W_12_O_42_]^10−^ | 0.29 ± 0.01 μM | - | [28] |
| BChE | [H_2_W_12_O_42_]^10−^ | 0.57 ± 0.09 μM | - | [28] |
| Aβ | POM-Dawson | 26.3 ± 4.32 μM | 4.97 μM | [29] |
| Aβ | POMds-Dawson-Co | 14.7 ± 2.37 μM | 1.10 μM | [29] |
| Aβ | POMds-Dawson-Ni | 5.6 ± 1.53 μM | 0.595 μM | [29] |
